# Examination of Surface Deposits on Oldbury Reactor Core Graphite to Determine the Concentration and Distribution of ^14^C

**DOI:** 10.1371/journal.pone.0164159

**Published:** 2016-10-05

**Authors:** Liam Payne, Peter J. Heard, Thomas B. Scott

**Affiliations:** Interface Analysis Centre, University of Bristol, Bristol, BS8 1TL, United Kingdom; University of Liverpool, UNITED KINGDOM

## Abstract

Pile Grade A graphite was used as a moderator and reflector material in the first generation of UK Magnox nuclear power reactors. As all of these reactors are now shut down there is a need to examine the concentration and distribution of long lived radioisotopes, such as ^14^C, to aid in understanding their behaviour in a geological disposal facility. A selection of irradiated graphite samples from Oldbury reactor one were examined where it was observed that Raman spectroscopy can distinguish between underlying graphite and a surface deposit found on exposed channel wall surfaces. The concentration of ^14^C in this deposit was examined by sequentially oxidising the graphite samples in air at low temperatures (450°C and 600°C) to remove the deposit and then the underlying graphite. The gases produced were captured in a series of bubbler solutions that were analysed using liquid scintillation counting. It was observed that the surface deposit was relatively enriched with ^14^C, with samples originating lower in the reactor exhibiting a higher concentration of ^14^C. Oxidation at 600°C showed that the remaining graphite material consisted of two fractions of ^14^C, a surface associated fraction and a graphite lattice associated fraction. The results presented correlate well with previous studies on irradiated graphite that suggest there are up to three fractions of ^14^C; a readily releasable fraction (corresponding to that removed by oxidation at 450°C in this study), a slowly releasable fraction (removed early at 600°C in this study), and an unreleasable fraction (removed later at 600°C in this study).

## Introduction

Graphite was used as a moderator and reflector material in the UK Magnox gas-cooled reactors due to its low capture and high scattering cross sections [1, 2]. However, fast neutron bombardment during operational lifetime resulted in the formation of several activation products [3] and therefore a suitable radioactive waste management strategy is required for the safe disposal of graphite waste [1]. In the United Kingdom approximately 45,000 m^3^ of Pile Grade A (PGA) graphite waste will be generated during the decommissioning of the first generation of gas-cooled, graphite-moderated Magnox reactors that requires disposal [4, 5]. The current UK baseline for this waste is classification as intermediate level waste (ILW) and disposal in a geological disposal facility (GDF) [4]. This classification is due to the presence of long lived radioisotopes, including a major proportion of ^14^C [6]. This radionuclide is significant for safety assessments of a GDF in the UK due to its long half-life (5730 years) [5] and its potential to form gaseous species that may be released after closure of a GDF [7].

Previous post mortem analysis of irradiated graphite from two Magnox reactor cores highlighted the presence of a carbonaceous deposit from one of the reactors [8]. This had a markedly different morphology to the bulk graphite on the exposed surface of the graphite bricks. The amounts of surface deposits observed here are likely to be the largest for the Magnox fleet due to the gas chemistry used in this reactor, and it is anticipated that not all Magnox reactors may contain such significant deposits. However, previous examination of these surface deposits using secondary ion mass spectrometry (SIMS) has determined that they have a relative enrichment of ^14^C compared to the bulk graphite [9]. The behaviour of these deposits in a GDF environment is not thoroughly understood as there is likely to be a difference in the behaviour of the deposits and the graphite which it coats due to the different reactivity of the materials [10]. Specifically, the deposit may contribute significantly to the release rate and magnitude of the labile ^14^C fraction [11], which is expected to be released relatively early in the lifetime of a GDF [7].

### ^14^C formation

^14^C is generated in irradiated graphite due to neutron capture by either ^14^N, ^13^C or ^17^O, Eqs (1)–(3) respectively. However, the contributions from each precursor species are likely to differ due to variations in isotopic abundance and capture cross section, Table 1. The contributions from each have been examined in the mass balance study of Wylfa Reactor 1 by Metcalfe and Mills [12], where it was found that for Wylfa in particular, the C-14 precursor C-13 makes a more significant contribution to the total activity than N-14 at 10 wppm. Although it must be stated that this is for Wylfa and not Oldbury as studied in this work and previous work has shown significant differences in these two reactors [8]. The low isotopic abundance and low cross section of ^17^O suggests graphite arising from Magnox reactors is unlikely to contain any significant concentrations of ^14^C arising from this route [13]. ^13^C is a stable minor isotope of carbon and is therefore intrinsically present in graphite material. Therefore ^14^C arising from this is more likely to be distributed homogeneously throughout the graphite [14]. Throughout the manufacture and assembly of the nuclear graphite air would have naturally adsorbed onto the surface and introduced nitrogen impurities [14] of up to 10 ppm [15]. The availability of nitrogen would have also been increased due to air ingress during depressurisation as a result of routine shut-downs. Therefore, ^14^C arising from ^14^N is likely to be inhomogeneously distributed on the surface of the graphite, including not only the outer geometrical surface but the total surface present in the extensive pore structure [15]. The exact contributions from each precursor species is not known but research by Black *et al.* [16] showed that at nitrogen concentrations of greater than 10 ppm it is ^14^N that is the dominant production pathway compared to ^13^C.

$$\begin{array}{c} {}_{7}^{14}\text{N}\;+{\;}_{0}^{1}\text{n}\;\to {\;}_{6}^{14}\text{C}\;+{\;}_{1}^{1}\text{p} \end{array}$$ (1)

$$\begin{array}{c} {}_{6}^{13}\text{C}\;+{\;}_{0}^{1}\text{n}\;\to {\;}_{6}^{14}\text{C}\;+\;\gamma \end{array}$$ (2)

$$\begin{array}{c} {}_{8}^{17}\text{O}\;+{\;}_{0}^{1}\text{n}\;\to {\;}_{6}^{14}\text{C}\;+{\;}_{2}^{4}\text{H}{\text{e}}^{2+} \end{array}$$ (3)

**Table 1:** Properties of ^14^C precursor species [14].

Species	Isotopic abundance (%)	Capture cross section (barns)
^13^C	1.07	0.0015
^14^N	99.63	1.8
^17^O	0.04	0.235

### Thermal treatment

Lifetime monitoring of the Magnox reactor fleet has yielded an inventory of small, cylindrical graphite trepan samples which have been examined to aid in extension of generation lifetime [17]. Such samples can be subsequently examined to determine the distribution and concentration of ^14^C, however standard non-destructive radiation measurements of ^14^C in graphite are limited due to the self shielding nature of the graphite material. Therefore, an alternative method is required that can measure the total ^14^C concentration of these samples, which has previously involved the use of thermal oxidation combined with liquid scintillation counting (LSC) [1, 14, 18–23].

The work presented here uses a similar method that actively oxidises the graphite in a controlled manner to selectively remove the ^14^C from the different fractions of irradiated graphite, similar to work performed by the National Nuclear Laboratory (NNL) [24]. For this a thorough understanding of the oxidation behaviour of the graphite is required, and this has previously been studied on virgin PGA graphite [25]. This study highlighted that PGA graphite exhibits the three regimes of thermal oxidation that have been observed on various other types of nuclear graphite [26–28]. The one of most relevance for this work was the low temperature (<600°C) chemical rate regime. In this regime the oxidation rate is slow and relatively uniform, allowing the oxidising species to penetrate deep within the pore network of the graphite. Such deep penetration of the gases means that the oxidation is uniform over the total exposed surface of not only the outer geometrical surface but also within the pores. As ^14^C arising from the different precursors is believed to be located in different areas this would allow oxidation of ^14^C located on the pore surface area in preference to that located in the bulk graphite. In addition to this, oxidation at even lower temperatures has the potential to remove the surface deposit and any associated ^14^C without significantly oxidising the underlying graphite, as these deposits are not graphite and are more chemically reactive to air than the underlying graphite [10, 29].

In summary, this work aims to examine a selection of irradiated graphite samples from Oldbury reactor one to determine the activity and distribution of ^14^C.

## Materials and methods

### Sample provenance

Irradiated graphite samples from the reactor core at Oldbury reactor one, South Gloucestershire, were supplied by NNL with assistance from Magnox Ltd. These samples were selected from the archived material stored at the NNL and can be split into two major groups; those originating from a channel wall, i.e. where one face of the sample had been exposed to the channel, and those samples originating from the inner brick region of the graphite. Virgin PGA graphite analysed in this study was provided by Magnox Ltd., arising from surplus material from the construction of the reactor cores. The virgin samples were prepared by cutting cylinders using a 12mm coring drill which were then cut into 7mm sections using a South Bay Technology Inc. Model 650 low speed diamond cutting wheel with deionised water used as coolant.

### Raman characterisation

Raman spectra were obtained using a Renishaw Raman System 2000 instrument. Specimens were mounted under a Leica optical microscope and excited with 633 nm wavelength argon ion laser light. A fifty times objective lens was used on the system to maximise the signal obtained. Spectra were acquired from 10 areas in the Raman shift range 1000 to 3000 cm^-1^, with each individual spectrum being compiled from a cumulation of 10 scans. The 520 cm^-1^ peak from a silicon specimen was used for calibration.

### Thermal oxidation with liquid scintillation counting

#### Experimental apparatus

**Fig 1:**
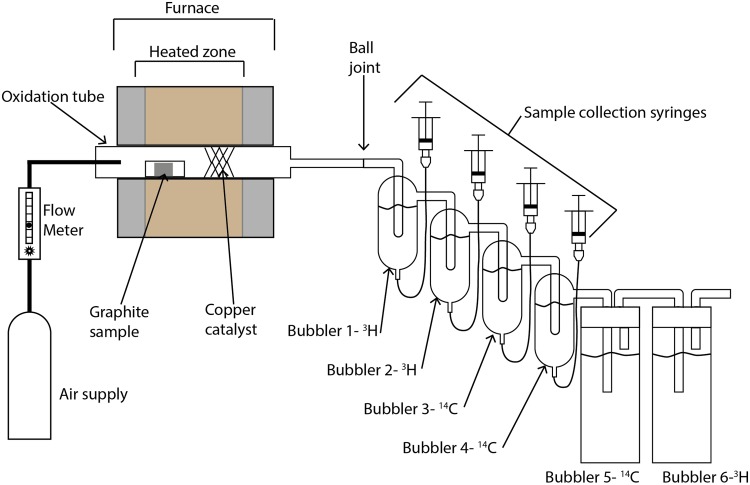
Labelled schematic of experimental apparatus used in the thermal oxidation of irradiated graphite.

The experimental apparatus for thermal oxidation is shown in Fig 1. A Carbolite HST 12/200 horizontal split tube furnace was used with a custom made quartz glass oxidation tube. The oxidation tube contained the sample followed by a copper catalyst, which was replaced for each sample to ensure consistent catalytic activity between samples. Gases produced in the oxidation tube flowed out of the outlet and entered a custom made bubbler system. This consisted of six bubblers, three designed to capture tritium and three designed to capture ^14^C. The tritium bubblers were used to minimise radioactive gas release. The first four bubblers were constructed as a single apparatus to minimise potential gas leaks while the last two were conventional bubblers present as a safety feature. The bubblers were numbered 1—6, with 1 being closest to the outlet of the oxidation tube and 6 being furthest away from the oxidation tube. Bubblers 1 and 2 contained 20 mL of 0.1 M HNO_3_ used for capturing tritium and bubblers 3 and 4 contained 20 mL of Carbo-Sorb^®^ E for capturing ^14^C. Bubblers 5 and 6 contained 100 mL of Carbo-Sorb^®^ E and 0.1 M HNO_3_ respectively.

During the experiment aliquots were taken by removing 2 mL of the solution, which was replaced with 2 mL of fresh solution, and mixing the aliquot with 2 mL of ScintLogic U scintillation cocktail (LabLogic) in a plastic scintillation vial prior to analysis using LSC. It was found that this 1:1 ratio of solution and cocktail were compatible and gave colourless solutions that reduced possible interferences in counting. Analysis for ^14^C was performed using a Hidex triathler LSC with a counting time of 60 minutes. A counting window of between 20 and 200 keV was used to minimise counts arising from ^3^H.

#### Experimental method

Each sample studied underwent two sequential experimental runs, firstly at 450°C and secondly at 600°C. Prior to oxidation the mass of the sample was recorded using an AB204-S analytical balance (Mettler Toledo). Raman spectroscopy was also performed as described above. After the initial weighing and Raman spectroscopy the sample was placed in the oxidation tube and a gas flow of 50 mL/min air introduced. The furnace was heated to 450°C at a rate of 30°C/min where it was held for a total duration of 50 hours.

2 mL aliquots were taken from bubbler 3 for ^14^C analysis at set intervals of 0, 1, 2, 3, 5, 8, 10, 25, 35 and 50 hours. Initially aliquots were also taken from bubbler 4 however after the first experimental run it was found that this was not necessary as the majority of the activity was captured in the first bubbler designed to capture ^14^C. At the end of the 50 hours 2 mL samples were also taken from the rest of the bubblers to determine the total radionuclide concentration, although the majority of the activity was captured in bubbler 3. The solutions were then removed from the bubblers and the bubblers rinsed and dried.

Once the sample had cooled sufficiently it was reweighed and Raman spectra recorded from the surface. The sample was then returned to the furnace and the bubbler solutions refilled with fresh solutions. Air was flowed through at 50 mL/min and the furnace heated to 600°C at a rate of 30°C/min where it was held for a total duration of up to 145 hours. The 2 mL aliquots taken for this experiment were taken at 0, 1, 2, 3, 5 and 7 hours as well as intervals up to the final duration. The total duration was determined by visual inspection of the oxidation tube and the experiment stopped when there were no visible remnants of the sample remaining. At the end of the run 2 mL samples were also taken from the rest of the bubblers. All apparatus were rinsed and catalyst and solutions replaced prior to the next sample.

In total five samples were analysed, one virgin PGA sample and four irradiated samples. The four irradiated samples were all from the same fuel channel, **Q15C5**, located in roughly the central region of the reactor at varying heights, **2U, 7L and 11U** equating to approximately 1.2, 5.1 and 8.5 m from the base of the reactor. Three of the four were channel wall face samples that had a deposit present and one (**Q15C5 2U Slice 2**) was an inner brick sample without a surface deposit.

#### Data analysis

The efficiency of the counter, specifically for the solutions analysed, was determined by preparation of a calibration sample made up in the same solution as the solutions analysed, i.e. 2 mL of Carbo-Sorb^®^ E mixed with 2 mL of ScintLogicU. The ^14^C used was a NIST traceable ^14^C-cholesterol standard with an activity of 1.7 kBq provided by Perkin Elmer. The solid was dissolved in the analysis solution and counted for 60 minutes three times, giving an efficiency of 0.0988 (9.88%). This efficiency was used to determine the activity in Becquerels.

During sampling 2 mL of solution was removed and replaced with 2 mL of fresh solution leading to a dilution of the remaining solution at a ratio of 18 parts original solution to 2 parts fresh solution, or 9:1. Therefore, for each aliquot (apart from the first which was undiluted) a corrected value was calculated, Eq (4). Here the activity of the previous measurement is corrected for the dilution and subtracted from the current activity measurement, giving the activity arising between the measurements. These values were used to calculate the cumulative ^14^C activity over time. As the mass losses were also known this cumulative total could be used to calculate total activity per mass, more specifically Bq/gram.

$$\begin{array}{c} C=A_{1}-\left(0.9\times A_{0}\right) \end{array}$$ (4)

Where:

- *C* is the corrected ^14^C activity in Bq
- *A*_0_ is the previous ^14^C activity measurement in Bq
- *A*_1_ is the current ^14^C activity measurement in Bq

## Results and Discussion

### Raman spectroscopy

Raman spectroscopy has been widely used to examine the properties of nuclear graphite [30, 31]. Previous research on irradiated PGA graphite has shown that there is a difference in Raman spectra obtained from virgin PGA, irradiated graphite and surface deposits [8]. In this work Raman spectroscopy was evaluated as a method of differentiating between these three types of carbon samples to allow rapid determination of the sample type in subsequent analysis. Previous literature has studied PGA graphite, however there is limited literature on ex-reactor samples that have been irradiated and have the carbonaceous deposit, described in [8], and therefore a Raman study was required to ensure validity of measurements.

**Fig 2:**
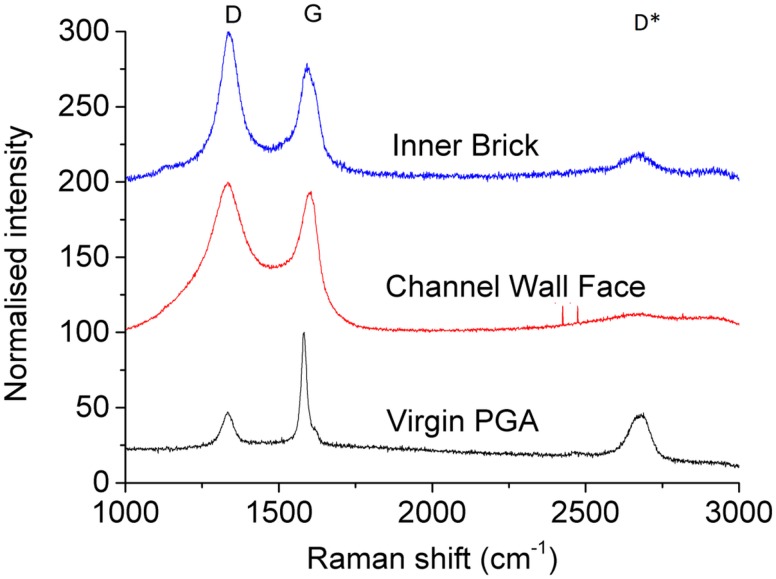
Normalised Raman spectra from irradiated (channel wall face and inner brick) and virgin PGA.

Prior to the examination of irradiated material, virgin PGA was examined to establish a baseline, so that any changes due to irradiation or deposition could be observed. The Raman spectra for unirradiated graphite has been widely reported and consists of two major peaks [32–34]. The most intense peak is at approximately 1580 cm^-1^ which is designated as the ‘G’ peak and is due to the E_2g_ mode of molecular vibration [35, 36]. The second peak, ‘D’, at approximately 1350 cm^-1^ is not present in ordered graphite, such as Highly Ordered Pyrolytic Graphite (HOPG), as it is only active in the presence of disorder. Tuinstra and Koenig [35] proposed this mode was an A_1g_ breathing mode that became Raman active due to a particle size effect with the intensity being dependant on the amount of ‘unorganised’ carbon in the samples and the graphite crystal size. A second-order peak at approximately 2700 cm^-1^ is also described, which corresponds to an overtone of the ‘D’ (disorder) mode and has been assigned many labels (G', 2D or D*) within the literature [31]. In this work it will be referred to as the D* peak. Raman spectra collected from virgin and irradiated PGA graphite are shown in Fig 2. The Raman spectrum from virgin PGA shows the three peaks described with the G peak being dominant.

The peaks observed from an inner brick surface were similar to those observed for virgin PGA, however there were some notable differences:

- A reduction in the intensity of the D* peak at approximately 2700 cm^-1^.
- An increase in intensity of the D peak at approximately 1350 cm^-1^.
- Significant increase in the width of both the D and G peaks.

These changes in Raman spectra are associated with an increased disorder within carbon material [37]. Within the irradiated graphite this increase in disorder is likely to be due to neutron irradiation induced microstructural changes, such as a reduction in graphite crystallite size, point defects and preferential loss of matrix material [8]. Raman spectra obtained from channel wall face surfaces differ from inner brick surfaces in two main ways:

- The D* peak at approximately 2700 cm^-1^ is not present due to a general rise in intensity at higher wavenumbers.
- There is further broadening of the D and G peaks.

It is suggested that the increase in peak width, especially the D peak, is due to an increase in disorder [38] and therefore a change in structure away from graphite towards an amorphous carbon.

The Raman spectra recorded can be split into three major groups; channel wall face samples with a deposit present, inner brick surfaces and virgin PGA. Examination of these sample types show some significant differences, most notably the widening of the D and G peaks. For true comparison a quantitative value for the extent of peak broadening is required. Peak fitting and calculation of the full width at half maximum (FWHM) was performed for this purpose using Origin software. The best fit was achieved using Lorentzian lines for both the D and G peaks which is in good agreement with the literature [31, 38, 39].

**Fig 3:**
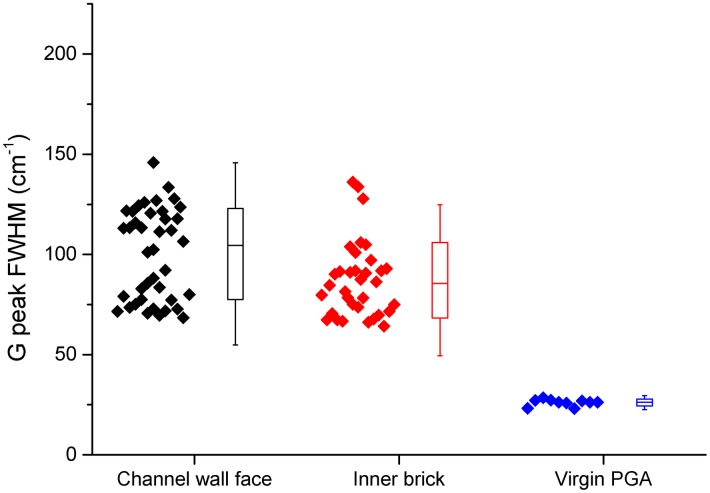
Box plot of G peak FWHM, box shows one standard deviation and lines show 2 standard deviations.

**Fig 4:**
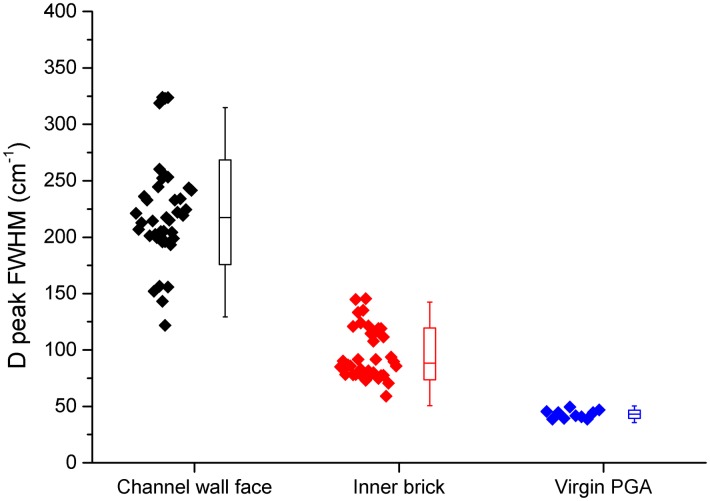
Box plot of D peak FWHM, box shows one standard deviation and lines show 2 standard deviations.

The calculated FWHM values for the G and D peaks for each of the three sample types are shown in Figs 3 and 4 respectively. Both the channel wall face samples and inner brick samples had significantly wider G and D peaks than virgin PGA, however there was only a significant difference in D peak FWHM for channel wall face and inner brick samples and not in the G peak FWHM. This further confirms that the Raman spectra arising from the channel wall face surfaces are due to the highly disordered deposited material. This significant difference in D peak FWHM allows Raman spectroscopy to be used, in conjunction with visual inspection, prior and post thermal oxidation at low temperatures to determine whether the deposited material has been removed.

### Thermal oxidation with LSC

The first experimental run was performed on virgin PGA to establish a baseline. Throughout the experiment the ^14^C concentration never increased above the value recorded for a blank sample (2 mL of Carbo-Sorb^®^ E mixed with 2 mL of ScintLogicU), approximately 90 Bq. The blank sample was subsequently counted before each sample run and this background value subtracted from the results obtained.

**Fig 5:**
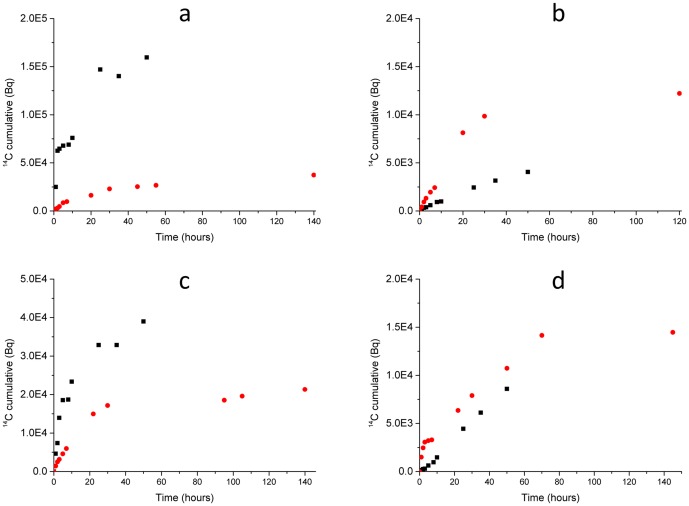
Cumulative ^14^C concentration for oxidised irradiated graphite samples at 450 (black) and 600 (red)°C. a) Channel wall face sample Q15C5 2U/1, b) Inner brick sample Q15C5 2U/2, c) Channel wall face sample Q15C5 7L/1 and d) Channel wall face sample Q15C5 11U/1.

The cumulative ^14^C activity released during oxidation at 450°C and 600°C for the four irradiated graphite samples are shown in Fig 5. The inner brick sample, Fig 5b, showed a relatively uniform and linear ^14^C release at 450°C with limited mass loss (0.9%). As there was no significant oxidation at this temperature the ^14^C must have arisen from the surface of the graphite, with the ^14^C present in the form of either deposited material similar to the surface deposit present in the pore structures or from surface adsorbed nitrogen that is not strongly bonded to the graphite that had been transmutated [14].

The channel wall face samples lower in the channel, Fig 5a and 5c, showed an initial, rapid release of ^14^C in the first few hours where it then reached an approximate plateau before increasing again between 10 and 25 hours. A final plateau was reached with a total activity significantly higher than the inner brick sample. The sample lowest in the channel, 2U, had a greater ^14^C activity than the sample higher up, 7L, which may be associated with a greater deposit mass, 5.3% compared to 2.4%. The remaining channel wall face sample was from the very upper region of the reactor core. The ^14^C release for this channel wall face sample was more similar to the inner brick sample than the other channel wall face samples, Fig 5d. This sample showed a relatively uniform release of ^14^C with the similarity to the inner brick sample believed to be due to a thin surface deposit, with only 1.6% of the total mass lost after 50 hours.

For samples Q15C5 2U Slice 1 and Q15C5 7L Slice 1 the ^14^C appears to be released as two fractions, the first a rapid initial release followed by a later release. The initial release is believed to be due to the release of ^14^C in the surface deposit, which was anticipated to occur very rapidly (within the first few hours). The cause of the secondary release is unknown but could potentially be due to a proportion of the deposit being protected by overlying deposited material, so that oxygen could not immediately react with this deposit, giving a delay in ^14^C release. It could also be from ^14^C formed from adsorbed ^14^N on the graphite surface that was initially protected by the deposit.

**Fig 6:**
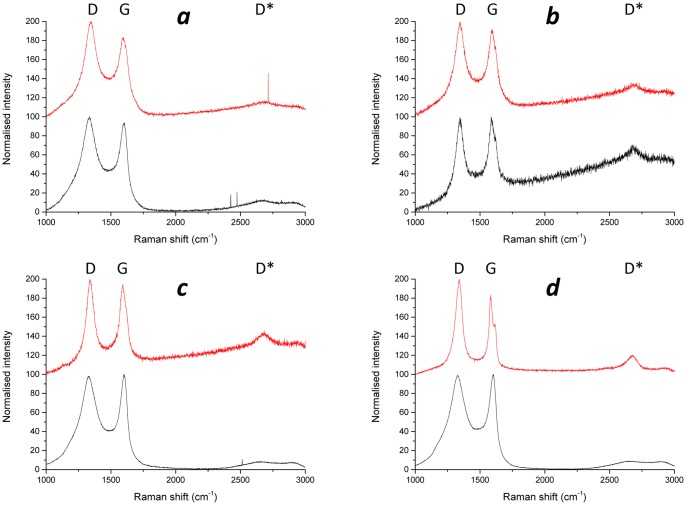
Normalised Raman spectra for irradiated graphite samples before (black) and after (red) oxidation at 450°C. a) Channel wall face sample Q15C5 2U/1, b) Inner brick sample Q15C5 2U/2, c) Channel wall face sample Q15C5 7L/1 and d) Channel wall face sample Q15C5 11U/1.

The mass losses suggested that the deposit was totally removed after oxidation for 50 hours at 450°C. Visual inspection and Raman spectroscopy confirmed the removal of the deposit. The Raman spectra from the four irradiated samples both prior and post oxidation at 450°C are shown in Fig 6. Prior to oxidation the samples that had surface deposits present had Raman spectra that showed increased D peak width assigned to surface deposits as described above. The inner brick sample gave spectra that were similar to other inner brick samples. Post oxidation all of the spectra were very similar, with the three samples that had showed the broad peaks associated with surface deposits now exhibiting peaks that were narrower, Tables 2 and 3. The spectra from the inner brick sample remained largely unchanged. Additionally the D* was present in the post oxidation spectra. These observations lead to the conclusion that the deposit that was present had been removed as the spectra recorded post oxidation were consistent with irradiated graphite.

**Table 2:** D peak width from Raman spectra pre and post oxidation at 450°C.

Sample	D peak FWHM pre-oxidation	D peak FWHM post-oxidation
Q15C5 2U Slice 1	205	130
Q15C5 2U Slice 2	40	65
Q15C5 7L Slice 1	181	51
Q15C5 11U Slice 1	180	64

**Table 3:** G peak width from Raman spectra pre and post oxidation at 450°C.

Sample	G peak FWHM pre-oxidation	G peak FWHM post-oxidation
Q15C5 2U Slice 1	88	85
Q15C5 2U Slice 2	43	69
Q15C5 7L Slice 1	68	50
Q15C5 11U Slice 1	70	64

The ^14^C release from oxidation at 600°C for each of the four irradiated graphite samples show a very similar pattern with an initial rapid release followed by a slower release and eventual plateau. Since any surface deposit present would have been removed at 450°C the similarities in the ^14^C release at 600°C between samples is not surprising since all samples would now have a similar structure.

As oxidation at 600°C will uniformly oxidise the surface of the graphite, the initial release of ^14^C observed suggests a significant proportion of the ^14^C is located on the surface (or near-surface) of the graphite, including all pore surfaces and not just the outer geometrical surface. The ^14^C located on the surface was likely formed from species adsorbing on the surface that originated as impurities from either the graphite core assembly or the coolant gas. Even though it is not possible to definitively determine the formation pathway of the ^14^C, this relatively large concentration on the surface strongly suggests formation from ^14^N which was known to be present in the coolant gas. Previous work by Dunzik-Gougar *et al.* [14] suggested that there may be substitution of single nitrogen atoms into displaced carbon atom locations in the surface and near surface of irradiated graphite that could explain the initial high release of ^14^C, however this work was performed to demonstrate nitrogen interactions with the graphite surfaces at extreme conditions and does not resemble the conditions found in a Magnox reactor. If such nitrogen complexes were present they would be bounded to the graphite and therefore not removed at 450°C but as the graphite material was removed at higher temperatures the ^14^C arising from this transmutated nitrogen would be released. After the early release (approximately 20 hours), the release rate decreases until it plateaus when all graphite material had been oxidised. This slower ^14^C release is representative of ^14^C that was present within the graphite structure and possibly graphite lattice. This fraction of ^14^C is believed to be formed from naturally occurring ^13^C and ^14^N impurities that were present within the graphite material and are released as the graphite oxidises.

**Fig 7:**
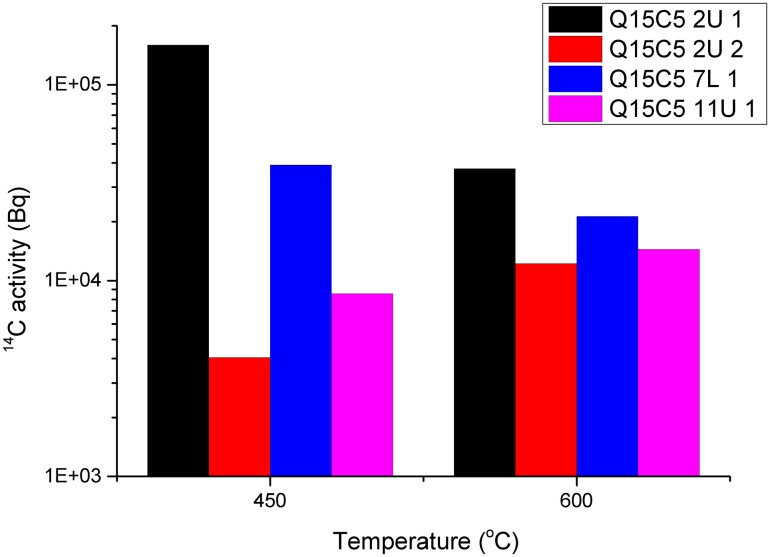
Total ^14^C activity release from irradiated graphite samples oxidised sequentially at 450°C and 600°C.

**Fig 8:**
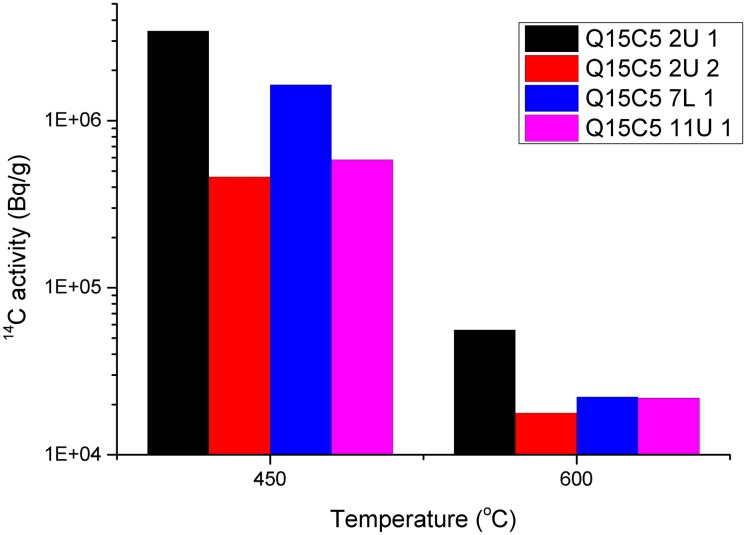
Cumulative ^14^C activity release normalised to mass loss from irradiated graphite samples oxidised sequentially at 450°C and 600°C. Log_10_ vertical scale is used for ease of visualisation.

The total activity of ^14^C in sample Q15C5 2U 1 was greater than Q15C5 7L 1, Fig 7, however this was potentially due to the different masses of deposit removed. When the activity is normalised to mass (Bq/g), Fig 8, the same pattern was observed, with Q15C5 2U 1 having a greater activity released at 450°C than Q15C5 7L 1 which in turn was greater than Q15C5 11U 1 which was greater than Q15C5 2U 2. A similar pattern was observed at 600°C although it was observable that all the activities per mass were lower at this temperature compared to 450°C.

The main interest in this work was the surface deposits on the channel wall face samples from Oldbury reactor one as previous analysis using SIMS [9] showed that these were relatively enriched in ^14^C compared to the underlying graphite. Therefore three samples with surface deposits were examined along with one inner brick sample without any detectable surface deposit. The samples chosen all originated from the same fuel channel, Q15C5, but at different heights that would allow relative ease of comparison. The heating of the inner brick sample (Q15C5 2U Slice 2) at 450°C showed that there was still a significant amount of ^14^C uniformly released, which initially was not expected as there was no detectable surface deposit. The exact origin of this ^14^C could not be accurately determined. However, it is believed to be a combination of adsorbed nitrogen species on the graphite surface that are only weakly bound and not bonded to the graphite lattice [14], and precursors that are substituted in to the graphite lattice in the very top layer of the graphite (first few nm) [40].

The channel wall face samples examined all showed an increase in both the total activity and total activity normalised to weight loss compared to the inner brick sample, although there were differences in the patterns of ^14^C release observed. The results obtained, especially at 450°C, correlate well with previously presented results collected using SIMS [9]. The activity determined by LSC in Bq was converted to ppm to aid in comparison to SIMS results. Comparison of the concentrations determined by SIMS and LSC, Table 4, shows that the results obtained by the two different methods are in very good agreement.

**Table 4:** Comparison of results from the concentration calculation, ppm, of ^14^C in surface deposits using LSC and SIMS results presented in [9]. Errors shows one standard deviation.

Sample	^14^C concentration SIMS (ppm) [9]	^14^C concentration LSC (ppm)
Q15C5 2U Slice 1	20.8 ±3.9	20.9 ±1.1
Q15C5 2U Slice 2	4.1 ±3.4	2.8 ±0.2
Q15C5 7L Slice 1	13.2 ±3.9	9.9 ±0.5
Q15C5 11U Slice 1	5.3 ±1.8	3.5 ±0.2

After full oxidation of the graphite there isn’t such a noticeable difference in ^14^C concentrations as observed after heating at 450°C, although Q15C5 2U Slice 1 had the highest activity followed by Q15C5 7L Slice 1, Q15C5 11U Slice 1 and finally Q15C5 2U Slice 2. These differences do not appear to be correlated with total neutron dose data provided by NNL. A possible explanation for the variation in ^14^C is the availability of precursor species in the coolant gas for the formation of surface species i.e. those not deep within the graphite lattice. During operation the coolant gas was pumped from the base of the reactor upwards, therefore the graphite lower in the channel would have been the first to be exposed to the gas. Therefore, any ^14^N or ^17^O present could adsorb onto the surface and form surface complexes [14] preferentially to graphite higher in the channel. This would lead to a greater concentration of precursor species bound to the surface of the graphite lower in the channel that could subsequently be converted to ^14^C. Temperature variations within the reactor core could also explain variation in the ^14^C concentrations as the temperature lower in reactor was less than higher regions, so that any ^14^C formed on the surface in lower regions was less likely to be discharged through gaseous pathways during operation. Similar results were observed by Metcalfe *et al.* [24], who showed that the deposit present on Oldbury graphite had a higher specific activity of ^14^C than the underlying graphite and that samples that originated lower in the channel had a higher ^14^C concentration. However, the authors did not postulate a reason for the relative enrichment in ^14^C in the surface deposits.

The results here correlate well with data presented by Baston *et al.* [11], who studied the release of ^14^C from Oldbury reactor 2 graphite when immersed in high-pH solution to simulate disposal in a GDF. During the study the authors examined the gaseous ^14^C released as well as ^14^C released into solution. Even though this study was on leaching behaviour, rather than ^14^C release during thermal oxidation, the authors developed a model where the ^14^C could be split into three fractions that may be useful in describing the results presented here. These three fractions are:

- A small, rapidly releasable fraction that is loosely bound to the graphite surface.
- A slowly releasable fraction that is located within the graphite pore structure.
- An essentially non-releasable fraction that is bound to the graphite structure.

The results presented in the present work show a fraction of ^14^C released at 450°C that represents the rapidly releasable fraction that is loosely bound to the graphite surface, largely made up of the surface deposit on the channel wall face along with adsorbed material on the surface of the graphite. Furthermore, the samples studied were small and may not be representative of the whole graphite core. Therefore, there are challenges in comparing the results quantitatively. However the presence of this fraction is important as it was observed even in inner brick samples without any detectable surface deposit, and the results obtained here are consistent with the findings of Baston *et al.* [11].

The slowly releasable fraction described matches well with the early initial release observed when the graphite was oxidised at 600°C. In this work the initial release was believed to be due to the incorporation of precursor molecules, mainly nitrogen, in graphite lattice vacancies that were formed due to irradiation damage. As the impurities were delivered in the coolant gas the ^14^C formed would be found on the graphite surface, however more strongly bonded than the adsorbed layer removed at 450°C. It is believed that this surface ^14^C would be removed slowly in a GDF environment as it is less reactive than the material removed at 450°C and is therefore believed to be the slowly releasable fraction described by Baston *et al.* The ^14^C released slowly after the initial release at 600°C when there is significant oxidation of the graphite is believed to be the non-releasable fraction described. This ^14^C would be incorporated into the graphite lattice and without a means of breaking down the graphite material, such as thermal oxidation, would never be released. Similiar conclusions were drawn in other leaching studies performed by Baston *et al.* [7] and Handy [41]. von Lensa *et al.* [40] (as part of the multi-national CARBOWASTE project) also studied the disposal behaviour of irradiated graphite and tentatively suggested that a proportion of the ^14^C inventory may be removed more easily than the rest, which is indicative of the ^14^C released at 450°C observed in this work.

## Conclusions

This work examined a series of irradiated graphite samples from Oldbury reactor one using a variety of techniques to investigate the concentration and distribution of ^14^C. Initially samples were characterised using Raman spectroscopy where a surface deposit with pronounced morphology was observed on surfaces directly exposed to the fuel channel. This deposit material was distinguishable from virgin PGA and inner brick irradiated graphite both visually and using the FWHM of the D Raman peak. This surface deposit is of interest for geological disposal of irradiated graphite waste as it may contain a fraction of the labile ^14^C. This work studied the use of thermal oxidation to selectively remove the surface deposit found on channel wall face graphite samples. This was to measure the ^14^C released from the surface and then subsequently fully oxidise the graphite to determine the total ^14^C concentration in the graphite. This was achieved by heating the graphite sample in a flow of air initially at 450°C and then at 600°C. In total four irradiated graphite samples from the same fuel channel were examined, one inner brick sample without a detectable surface deposit and three channel wall face samples with surface deposits from varying heights. Through this examination the following conclusions can be drawn:

- The release of ^14^C from the inner brick sample at 450°C was approximately linear and is suggested to have arisen from adsorbed nitrogen on the surface and subsurface of the graphite that is only weakly bound to the surface.
- The release of ^14^C from the two lower channel wall face samples at 450°C showed an initial rapid release followed by a slower release. The rapid release was determined to be due to ^14^C present in the surface deposit and the secondary release due to the adsorbed nitrogen that was initially protected by the deposit.
- The release of ^14^C from the upper channel wall face sample at 450°C was approximately linear and did not exhibit the initial rapid release observed in the lower channel wall face samples. This was because the deposit was very thin and the ^14^C is suggested to have arisen from the surface deposit and adsorbed nitrogen on the surface. There is believed to be a contribution from the surface deposit as the total activity was significantly higher than the inner brick sample.
- All samples showed a similar release pattern at 600°C with an initial rapid release followed by a slow, relatively uniform release. These two fractions were postulated to be due to nitrogen surface complexes on the graphite surface from the coolant gas and precursors(^13^C and ^14^N) present in the graphite lattice or as interstitial atoms respectively.
- The results of ^14^C release from surface deposits correlated well with previously reported results obtained using SIMS i.e. the surface deposit is relatively enriched in ^14^C compared to underlying graphite with samples lower in the channel showing a greater enrichment.

However, the results presented are only from a small fraction of the samples held at the University of Bristol, which are only a small fraction of the total graphite arising from Oldbury reactor one. Therefore, great care must be taken when using these results as they may not be representative of the material throughout the reactor. Also, radiolytic oxidation of the graphite and deposit would have continuously removed material, and associated ^14^C, during operation and this needs to be accounted for in any future modelling studies. Furthermore, the mass of the deposit compared to the mass of the sample would be much greater in the trepan samples studied here than in a whole graphite block. However, the method described here has shown good results and could be used for examination of graphite arising from other graphite moderated reactors.
